# Older immigrants’ use of general practice in Denmark a register-based cohort study

**DOI:** 10.1080/02813432.2026.2666625

**Published:** 2026-05-11

**Authors:** Amanda Luna Frank Strobel, Jesper Lykkegaard, Dorthe Susanne Nielsen, Jonas Kanstrup Olsen

**Affiliations:** ^a^ Research Unit of General Practice, Department of Public Health, University of Southern Denmark, Odense, Denmark; ^b^ Migrant Health Clinic, Odense University Hospital, Odense, Denmark; ^c^ Center for Global Health, University of Southern Denmark, Odense, Denmark

**Keywords:** Aged, general practice, primary health care, ethnic and racial minorities, emigrants and immigrants

## Abstract

**Background:**

Health care systems in Northern Europe are facing increasing numbers of older immigrants. This study aimed to examine older immigrants’ use of daytime general practice in Denmark compared with natives.

**Methods:**

In this register-based cohort study, all citizens aged ≥75 years were grouped into country categories according to four characteristics of their country of birth, including geographic region, gross national income, health expenditure and population density. Frequencies of face-to-face, telephone, email and home visit consultations among immigrants were compared with natives using zero-inflated Poisson regression, adjusted for individual health, geographic and socioeconomic factors.

**Results:**

Immigrants from countries with a low percentage of gross domestic product allocated to health care, as well as immigrants from middle-income countries (e.g. Pakistan (incidence rate ratio (IRR) 0.83 (95% confidence interval (95% CI) 0.82; 0.85)) and Bosnia and Herzegovina (IRR 0.82 (95% CI 0.80; 0.83))), generally had fewer consultations in general practice compared with natives. In contrast, immigrants from high-income countries and from countries with high health care spending had more consultations than natives. Seven country categories were identified based on the four characteristics, with low-income countries not represented in any category as they did not meet the threshold of 500 immigrants per category.

**Conclusion:**

Among older immigrants in Denmark, the use of services in daytime general practice is closely associated with the health care expenditure in the country of birth.

## Background

General practice in the Northern European health care systems is undergoing a demographic transition, with increasing numbers of older immigrants, many of whom arrived decades ago and are now ageing [1]. Most immigration to Scandinavian countries has occurred during the last 50 years and has primarily consisted of migrant workers and asylum seekers, the majority of whom were young and healthy at the time of entry [2,3].

The provision of health care for immigrants is challenged by linguistic, psychological and sociocultural differences [4,5]. Furthermore, non-western immigrants may be at higher risk of diabetes, cardiovascular diseases and mental health problems [6], as well as communicable diseases, such as tuberculosis, hepatitis and HIV [7]. They may also present with conditions that are less commonly encountered by Danish general practitioners (GPs).

Ethnic minority populations are more likely to experience disparities in health outcomes and health care compared with native-born populations [8]. In coming decades, older immigrants will constitute an increasing share of patients entitled to the same services in general practice as older natives [9,10], although utilisation patterns are likely to differ.

Previous research has shown that some ethnic minority groups have higher utilisation rates of general practice, although patterns vary substantially across populations and health care systems. Differences in morbidity and access barriers, including language and ability to navigate services, may contribute to this heterogeneity [11–13]. However, evidence on consultation patterns among older immigrants from different countries remains limited. This is particularly relevant given its implications for allocating resources across general practices with varying proportions of immigrant patients and for mitigating potential inequities in health care [14].

This study aimed to examine older immigrants’ use of daytime general practice in Denmark compared with native-born older Danes.

## Methods

### Design, setting and population

We conducted a nationwide register-based cohort study. Denmark is a Scandinavian country with a population of approximately six million. Between 2018 and 2060, the proportion of immigrants and descendants in the population is expected to increase from 13.3 to 21.0% [15]. The majority of health care services, including general practice, are fully tax-funded. More than 98% of citizens are listed with a specific general practice [16]. GPs serve as the first point of contact with the health care system, provide primary and preventive care, and act as gatekeepers to further specialised services [16,17]. Denmark has ∼1600 general practices, which operate either as solo or group practices [18]. On average, older patients have been registered with their current general practice for 9.5 years [19]. Until 2018, professional interpretation services in the Danish health care system were provided free of charge. Since then, immigrants who have resided in Denmark for more than three years must pay a fee for these services.

The study included all citizens aged ≥75 years who, on their birthday in 2021, were listed with a Danish general practice. To minimise the influence of outliers and reduce bias related to practice turnover during the year, we excluded practices with fewer than 500 registered patients or 20 eligible patients at the beginning or end of 2021, as well as those without at least one remunerated consultation per month.

### Data sources

Data were linked using each resident’s unique personal identification number provided by the Danish Civil Registration System [20]. General practice services were obtained from the Danish National Health Service Register [21]. Links between patients and general practices were established using the Patient List Database. Data were obtained from several national registers, including The Danish Civil Registration System (sex, age, migration and vital status) [20], Statistics Denmark (marital status, income, address and home health care services) [22], the Danish National Patient Register (hospital admissions, including dates, International Classification of Diseases 10th revision (ICD-10) diagnosis codes and procedures) [23], the Danish Psychiatric Central Research Register (psychiatric hospital admissions and ICD-10 diagnosis codes) [24] and the Danish National Prescription Register (redeemed prescriptions, including Anatomical Therapeutic Chemical Classification codes) [25]. Data were obtained on country-level indicators from the World Bank Data Bank [26–28].

### Definition of variables

Statistics Denmark’s definition of immigrants was applied: ‘An immigrant is defined as a person born abroad whose parents are both (or one of them if there is no available information on the other parent) foreign citizens or were both born abroad’ [29]. Countries of origin were categorised into seven geographic categories, based on the Danish Health Authority [30]: Danish, other Western, Eastern European, Middle Eastern or Northern African, Sub-Saharan African, Central or Southern American and Asian or Oceanian background (Supplementary Material 1). Notably, countries, such as Slovakia and Poland, were classified as Western owing to their status as member states of the European Union. If a country of origin no longer existed (e.g. the Soviet Union), data from the present-day country most closely corresponding to the former state (e.g. Russia) were used (Supplementary Material 2).

Gross national income (GNI) per capita were categorised into low (<1085 USD), middle (1086–13,205 USD) and high-income (>13,205 USD) countries as defined by the World Bank [31].

To mitigate fluctuations and account for missing data, health care expenditure was averaged over a four-year period from 2017 to 2020. The expenditures were expressed as percentages of gross domestic product (GDP) and categorised into low (<6%), middle (6–10%) and high (>10%). Population density was classified as either low (<200 inhabitants per square kilometre) or high (≥200 inhabitants per square kilometre).

Broader geographic categories were applied to provide an overview and a pragmatic classification of countries, consistent with the classification applied by the Danish Health Authority. Country-level indicators were included to capture variations between countries and to stratify immigrants into categories with comparable country of origin profiles. Countries of origin were grouped based on all possible combinations of geographic region, GNI, health care expenditure percentage and population density. Each category of countries was labelled according to the country contributing the largest number of immigrants.

The Nordic multimorbidity index was used to classify levels of multimorbidity into none, low, middle or high [32]. To account for medical complexity and frailty, the number of prescription drugs was categorised into groups of 0, 1–4, 5–9 and ≥10 unique drugs. Municipality home health care services were divided into categories: none, lowest, middle, highest tertile and residence in a nursing home (Supplementary Material 3). Patients whose country of origin was unknown were excluded from the analyses. To exclude temporary local project services, consultation types performed fewer than 1000 times during 2021 were excluded. Remuneration codes used to categorise face-to-face, telephone, email and home visit consultations are provided in Supplementary Material 4.

### Statistical analysis

To focus on the most relevant categories of countries, combinations with fewer than 500 immigrants were excluded from the analyses.

Zero-inflated Poisson regression was used to analyse adjusted consultation rates for each combination. This model enabled estimation of the incidence rate ratio (IRR) for consultations among patients who attended general practice during the year, and estimation of the odds ratio (OR) for the association with non-utilisation of general practice.

To adjust for potential confounding and better isolate the association between country of origin and consultation rates, analyses were adjusted for health, geographic and socioeconomic factors that may influence general practice utilisation. Health factors (age, sex, multimorbidity, redeemed prescription drugs and level of home health care services) were included to account for differences in underlying morbidity and health care needs. Geographic factors (rural or urban district, travel distance from home address to general practice and travel distance to the nearest emergency department) were included because access to and availability of services may vary by place of residence [33]. Travel distances were calculated using the patient’s registered home address, allowing distance to be determined for all individuals regardless of whether they had received a home visit. Socioeconomic factors (cohabitation, household income and household wealth) were included because social gradients may affect health literacy and the ability to navigate the health care system, thereby influencing patterns of general practice use. Hierarchical sensitivity analyses were also conducted, in which models were sequentially adjusted for health, geographic and socioeconomic factors. All analyses were conducted at the patient level. Analyses were performed for both total consultations and separately for each consultation type. Statistical analyses were conducted using Stata 18.0.

## Results

### Patient characteristics and country of origin-related factors

The study included 532,867 patients aged ≥75 years, of whom 57 were excluded due to an unknown country of origin. Natives constituted 96% of the population, other Western immigrants 2.8%, Eastern European immigrants 0.31%, Middle Eastern/Northern African immigrants 0.68%, Sub-Saharan African immigrants 0.09%, Southern/Central American immigrants 0.09% and Asian/Oceanian immigrants 0.50%. Across these geographical groups, immigrants’ median ages ranged from 78 to 79 years. Immigrants from Middle Eastern/Northern African countries were more often male (57%), whereas those from other Western countries were less often male (40%). The median number of prescription drugs ranged from four to five, and the median level of multimorbidity ranged from two to four. Nursing home residency was lower among immigrants from Asian/Oceanian (2.5%) and Middle Eastern/Northern African (1.7%) countries compared with natives (4.0%).

Asian/Oceanian (52%) and Middle Eastern/Northern African (55%) immigrants had the highest proportions of cohabitation. Sub-Saharan African countries had the lowest median GNI per capita, while Middle Eastern/Northern African, Sub-Saharan African and Asian/Oceanian countries had the lowest percentages of health care expenditure. For complete results, see Tables 1 and 2.

**Table 1.** Patient characteristics by geographical origin among citizens aged ≥75 years in Denmark in 2021^a^.

Population	Denmark	Other Western	Eastern European	Middle Eastern/Northern African	Sub-Saharan Africa	Southern/Central American	Asian / Oceanian
*N* = 532,810	509,213	14,688	1694	3629	470	472	2644
Patient health factors							
Age (years)	79 (76–83)	79 (76–83)	79 (76–82)	78 (75–81)	78 (75–83)	79 (76–83)	78 (76–85)
Male sex (%)	43.6 (43.6–43.6)	40.2 (32.0–43.3)	41.9 (42.3–43.0)	57.0 (52.3–60.2)	53.2 (38.4–73.7)	40.7 (29.7–47.0)	47.3 (37.8–63.7)
Nordic multimorbidity index	3 (0–10)	3 (0–10)	3 (0–10)	4 (0–11)	3 (0–11)	2 (0–9)	3 (0–10)
Number of prescription drugs	4 (2–7)	4 (2–6)	5 (2–7)	5 (2–8)	4 (1–7)	4 (2–6)	4 (2–7)
Receiving home health care services (%)	12.39 (12.30–12.39)	11.97 (11.11–13.12)	10.68 (9.07–13.02)	13.89 (12.21–15.62)	13.62 (5.88–16.95)	10.59 (7.30–15.00)	11.91 (10.42–13.13)
Nursing home residents (%)	4.03 (4.03–4.03)	4.13 (3.45–4.67)	3.60 (2.36–3.57)	1.68 (1.44–2.07)	3.19 (0–6.78)	4.66 (1.92–5.11)	2.53 (1.24–3.66)
Patient geographic factors							
Rural district (%)	9.24 (9.24–9.24)	8.82 (7.72–10.84)	1.30 (1.37–1.81)	0.88 (0.08–2.27)	4.26 (2.26–8.33)	6.14 (3.65–10.00)	1.74 (0.85–1.96)
Travel distance to practice (km)	3 (1–6)	2 (1–5)	2 (1–3)	2 (1–4)	3 (1–5)	2 (1–4)	2 (1–4)
Travel distance to hospital (km)	16 (6–27)	13 (5–25)	6 (3–19)	6 (4–14)	6 (3–13)	8 (4–19.5)	6 (4–13)
Patient socioeconomic factors							
Cohabitating (%)	49.39 (49.39–49.39)	46.22 (43.25–47.93)	47.28 (45.21–46.84)	55.28 (49.48–58.23)	48.30 (45.76–48.02)	45.34 (41.61–46.15)	52.16 (44.92–63.19)
Household income per year (EUR)	27,887.05 (24,046.78–36,046.55)	28,893.19 (23,838.61–38,695.96)	22,281.08 (18,209.29–24,222.70)	21,487.76 (18,297.99–23,934.32)	22,993.41 (18,659.52–28,554.84)	25,431.90 (21,721.58–35,611.19)	22,068.36 (17,965.68–26,132.80)
Household wealth (EUR)	100,777.48 (30,529.76–232,787.53)	83,408.18 (20,315.48–225,945.17)	52,36.53 (1523.59–21,022.79)	5372.99 (1611.39–14,814.34)	7474.36 (1402.01–73,993.77)	57,710.86 (12,364.21–191,263.53)	11,435.59 (2912.20–82,919.70)

^a^ Population comprises all citizens aged ≥75 years living in Denmark in 2021. Geographical origin is defined by country of birth and grouped into predefined geographic categories.

Data are presented as medians, with parentheses indicate the 25th–75th percentile (p25–p75).

**Table 2.** Country of origin characteristics among citizens aged ≥75 years in Denmark in 2021^a^.

	Denmark	Other Western	Eastern European	Middle Eastern/Northern African	Sub-Saharan African	Southern/Central American	Asian/Oceanian
GNI per capita (USD)	69,780 (69,780–69,780)	52,050 (4550–80,860)	7050 (6910–8470)	4690 (3620–10,010)	860 (580–2280)	9980 (7880–14,760)	3290 (1470–4020)
Health expenditure of GDP (%)	10.2 (10.2–10.2)	11.0 (10.3–11.8)	8.5 (8.5–9.2)	4.4 (4.3–6.6)	4.1 (3.5–6.7)	9.3 (7.0–9.7)	3.9 (2.9–4.9)
Population density (population per ${\text{km}}^{2}$ )	146 (146–146)	123 (26–238)	64 (64–81)	109 (83–109)	49 (27–107)	32 (20–150)	300 (150–358)
Years since migration	n.a.	48 (45–48)	26 (25–48)	42 (30–48)	33 (23–48)	47 (41–48)	43 (31–48)

^a^ Population comprises all citizens aged ≥75 years living in Denmark in 2021. Geographical origin is defined by country of birth and grouped into predefined geographic categories.

Data are presented as medians, with parentheses indicate the 25th–75th percentile (p25–p75).

GNI: gross national income; GDP: gross domestic product; n.a.: not applicable.

### Total consultation frequency

**Figure 1.**
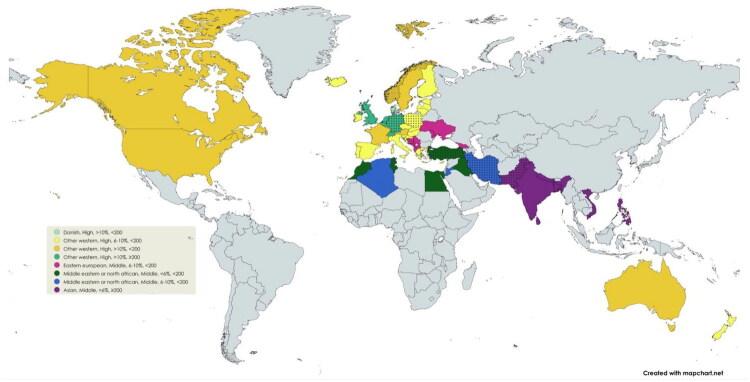
The figure illustrates all categories defined by combinations of country of origin characteristics comprising ≥500 immigrants in each category. The most prevalent country within each category is indicated by a dotted pattern. Order of characteristics: geographical region, income level, health expenditure and population density.

Combining the characteristics, seven country categories were created. Table 3 and Figure 1 show, for each category, the country contributing the largest number of immigrants. Low-income countries were not represented among the resulting country categories due to the insufficient number of immigrants from these countries for reliable analyses.

**Table 3.** IRR for total general practice consultations across categories stratified by characteristics of country of origin^a^ among citizens aged ≥75 years in Denmark in 2021.

Characteristics of country of origin	*N*	Most prevalent country (%)		*n*	Mean number of consultations	Median number of consultations^b^	IRR crude	95% CI	IRR adjusted^c^	95% CI
Denmark, high, >10%, <200	509,213	Denmark	100	509,213	10.06	7 (2–14)	Reference	Reference	Reference	Reference
Other Western, high, 6–10%, <200	3042	Poland	30	900	9.84	7 (2–14)	1.00	(0.99; 1.02)	1.02	(1.01; 1.03)
Other Western, high, >10%, <200	5006	Norway	38	1905	10.76	8 (2–15)	1.07	(1.06; 1.07)	1.07	(1.06; 1.08)
Other Western, high, >10%, ≥200	6473	Germany	70	4559	10.19	7 (2–15)	1.02	(1.02; 1.03)	1.04	(1.04; 1.05)
Eastern European, Middle, 6–10%, <200	1441	Bosnia and Herzegovina	51	728	7.42	4 (1–11)	0.82	(0.80; 0.83)	0.82	(0.80; 0.83)
Middle Eastern/Northern African, Middle, <6%, <200	2309	Türkiye	57	1324	7.88	5 (1–12)	0.87	(0.85; 0.88)	0.84	(0.83; 0.85)
Middle Eastern/Northern African, Middle, 6–10%, <200	635	Iran	75	475	9.27	6 (1–14)	1.01	(0.98; 1.03)	1.01	(0.99; 1.04)
Asian/Oceanian, Middle, <6%, ≥200	1772	Pakistan	41	728	7.20	4 (0–11)	0.84	(0.83; 0.85)	0.83	(0.82; 0.85)

^a^ Characteristics of country of origin refer to country-level attributes of each individual’s country of birth. These include geographical region, national income level, national health expenditure and population density, which together define the categories presented in the table.

^b^ Parentheses indicate the 25th–75th percentile (p25–p75).

^c^ Adjusted for age, sex, multimorbidity, polypharmacy, level of home health care services, rural or urban district, travel distance from home address to general practice, travel distance to appointed emergency department, cohabitation, household income and household wealth.

Order of characteristics: geographical region, income level, health expenditure and population density.

Low-income countries are not represented in the table, as the number of immigrants from these countries was insufficient to meet the inclusion criterion of ≥500 immigrants per category.

IRR: incidence rate ratio; 95% CI: 95% confidence interval.

**Figure 2.**
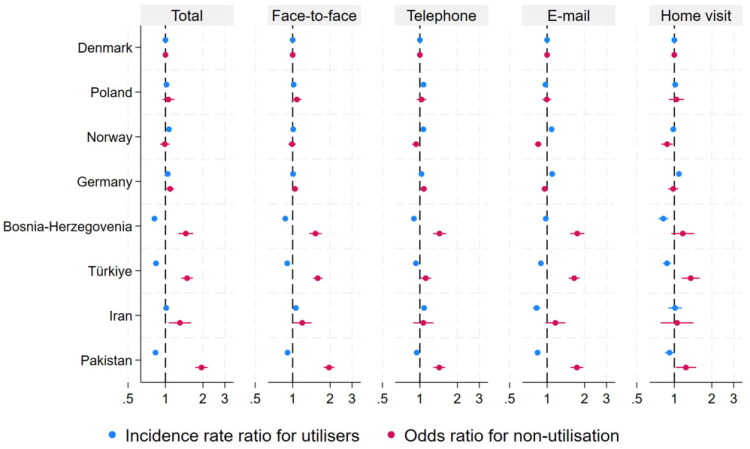
The figure displays the results of a zero-inflated Poisson regression for the total number of consultations and for consultations stratified by type among citizens aged ≥75 years living in Denmark in 2021^1^. A ratio >1 for incidence rate ratio indicates more total consultations compared with native Danes, adjusted for all other parameters*. A ratio >1 for odds ratio indicates a higher likelihood of non-utilisation of general practice, adjusted for all other parameters*. *Adjusted for age, sex, multimorbidity, polypharmacy, level of home health care services, rural or urban district, travel distance from home address to general practice, travel distance to appointed emergency department, cohabitation, household income and household wealth. ^1^See Supplementary Material 4 for remuneration codes.

Compared with natives, total consultation frequency was higher among immigrants in the Poland (IRR 1.02 (95% confidence interval (95% CI) 1.01; 1.03)), Norway (IRR 1.07 (95% CI 1.06; 1.08)) and Germany (IRR 1.04 (95% CI 1.04; 1.05)) categories (Figure 2). In contrast, total consultation frequency was lower among immigrants in the Bosnia and Herzegovina (IRR 0.82 (95% CI 0.80; 0.83)), Türkiye (IRR 0.84 (95% CI 0.83; 0.85)) and Pakistan (IRR 0.83 (95% CI 0.82; 0.85)) categories.

Immigrants from high-GNI per capita country categories generally had higher consultation frequencies, whereas those from middle GNI categories had lower frequencies, except for the Iran category. Consultation rates in general practice were lower among immigrants from countries with low health care expenditure (<6%), including the country categories Türkiye (IRR 0.84 (95% CI 0.83; 0.85)) and Pakistan (IRR 0.83 (95% CI 0.82; 0.85)).

No clear association was observed between the population density of the country of origin and immigrants’ total consultation frequencies. For complete results, see Table 3.

In the hierarchical sensitivity analyses, immigrants in the Norway category had higher consultation rates than natives across all models: crude (IRR 1.07 (95% CI 1.06; 1.08)), adjusted for health factors (IRR 1.08 (95% CI 1.07; 1.09)), adjusted for health and geographic factors (IRR 1.07 (95% CI 1.07; 1.08)) and fully adjusted for health, geographic and socioeconomic factors (IRR 1.07 (95% CI 1.06; 1.08)). A similarly consistent pattern was observed for immigrants in the Bosnia and Herzegovina, Türkiye and Pakistan categories, which showed lower consultation frequencies than natives across all hierarchical models. For complete results, see Supplementary Material 6.

When examining the mean and median number of total general practice consultations, older natives had a mean of 10.06 consultations per year. Immigrants in the Poland, Norway and Germany categories showed comparable tendencies. In contrast, mean total consultation counts were lower among immigrants in the Bosnia and Herzegovina (mean 7.42), Türkiye (mean 7.88) and Pakistan (mean 7.20) categories than among natives. This pattern was consistent across all consultation types. For complete results, see Supplementary Material 7.

### Frequency of consultation types

Immigrants in the other Western country categories, including Poland (IRR 1.07 (95% CI 1.05; 1.09)), Norway (IRR 1.07 (95% CI 1.05; 1.08)) and Germany (IRR 1.03 (95% CI 1.02; 1.04)), had higher rates of telephone consultations than natives.

Immigrants in the Iran category had higher utilisation of face-to-face consultations (IRR 1.06 (95% CI 1.02; 1.10)) and telephone consultations (IRR 1.08 (95% CI 1.04; 1.13)) compared with natives.

Immigrants in the Bosnia and Herzegovina, Türkiye and Pakistan categories had fewer consultations than natives across all consultation types, with few exceptions. For complete results, see Supplementary Material 5.

Across consultation types, immigrants in the Poland, Norway and Germany categories had mean and median consultation counts similar to those of natives, with few exceptions. Immigrants in the Bosnia and Herzegovina, Türkiye and Pakistan categories had lower mean consultation counts across consultation types. Full results are provided in Supplementary Material 7.

### Non-utilisation

Immigrants in the Poland and Norway categories did not have significantly different odds of non-utilisation compared with natives, although results differed for Norway for email consultations (OR 0.85 (95% CI 0.80; 0.90)) and home visits (OR 0.88 (95% CI 0.79; 0.97)). Immigrants in the Germany category showed increased odds of non-utilisation for total consultations, although results varied across consultation types. Immigrants in the Bosnia and Herzegovina (OR 1.57 (95% CI 1.30; 1.89)) and Türkiye (OR 1.49 (95% CI 1.34; 1.66)) categories had higher odds of non-utilisation than natives across all consultation types.

The highest odds of non-utilisation for total consultations were observed among immigrants in the Pakistan category (OR 1.96 (95% CI 1.74; 2.18)), with increased odds across most consultation types. Most immigrant categories showed increased odds of non-utilisation both for total consultations and across consultation types, with few exceptions. Full results are provided in Supplementary Material 5.

Across the Bosnia and Herzegovina, Türkiye, Iran and Pakistan categories, the odds of non-utilisation were lower in the fully adjusted model compared with the crude estimates. Full hierarchical results are presented in Supplementary Material 6.

## Discussion

### Main findings

Older natives had a mean of 10 general practice consultations per year, whereas some categories of older immigrants had as few as seven, for example, immigrants in the Bosnia and Herzegovina, Türkiye and Pakistan categories.

Consultation rates increased with the income level of the country of origin, with older immigrants from higher-income countries showing higher use of general practice than natives. In contrast, older immigrants from countries with lower health care expenditure had the fewest consultations and the highest odds of non-utilisation, regardless of adjustment for health, geographic and socioeconomic differences.

### Strengths and limitations

The main strength of this study is the nationwide population with almost complete register data at the individual level, enabling detailed analyses of consultation frequencies and adjustment for health, geographic, and socioeconomic factors. Nevertheless, some immigrant populations were small, necessitating the pooling of immigrants from different countries of origin. This study focused on the most vulnerable segment of the older population; therefore, analyses were limited to individuals aged ≥75 years. Younger older adults represent a more heterogeneous group, with some still active in the workforce. The chosen age group also reflects the current and projected increase in older patients linked to the post-World War II increased birth rates.

Furthermore, using GNI per capita to classify countries of origin into low-, middle- and high-income categories, as defined by the World Bank [31], is a strength, as it facilitates comparability with other research applying similar income classifications.

This study focused specifically on daytime general practice consultations, which represent the core of continuous primary care in Denmark. However, general practice-based out-of-hours services also represent an important component of primary care utilisation. These consultations were not captured in the present analysis, and their exclusion should be considered when interpreting the findings, as utilisation patterns may differ between daytime and out-of-hours services. Although some patients may seek out-of-hours primary care as a substitute for daytime general practice services, the two are not directly comparable. Regular investigations, such as blood sampling and spirometry, cannot be performed out-of-hours, and patients cannot be referred to imaging or to nonacute secondary or primary care service.

A limitation of the study arises from its use of the Nordic multimorbidity index to adjust for patients’ overall health and morbidity. By using this index based on hospital diagnoses and prescribed medication, it assumes equal access to hospital and general practice services across population groups. This assumption may not be accurate for immigrants, given our findings of lower consultation frequencies, and may lead to an underestimation of morbidity.

Immigrants whose country of origin no longer exists were classified as the country most closely resembling the former state, for example, Russia for the former Soviet Union (Supplementary Material 2).

The use of Statistics Denmark’s definition of immigrants may introduce bias, as this classification does not fully capture heterogeneity within immigrant populations or differences in migration history. Nevertheless, Statistics Denmark remains the most comprehensive and reliable source of national-level data currently available. Data used to classify country of origin were from 2021, as we were unable to obtain historical data corresponding to each immigrant’s time of immigration. This may have led to misclassification, as changes in national wealth or health care expenditure over time were not captured. Among the largest immigrant groups in Denmark, both Germany and Norway have had a marked increase in health care expenditure, whereas Pakistan and Türkiye have maintained relatively stable levels since 2000, when World Bank data collection began.

Some older immigrants may prefer to receive medical care in their country of origin rather than in Denmark. This is known as the salmon bias [34] and may be more relevant for immigrants from countries with well-developed health care systems, who may return to their country of origin for treatment. Such behaviour could lead to an underestimation of consultation frequency in Denmark and an overestimation of non-utilisation. This bias is likely less pronounced among immigrants from countries with limited medical infrastructure or access.

### Comparison to other studies

Several studies have reported that immigrants generally have better overall health than the native populations. This pattern is often attributed to the healthy immigrant effect, meaning that healthy and robust members of an origin population are more likely to migrate and therefore have less need for medical services in the host country [35,36]. In line with our findings, several international studies have documented reduced general practice attendance among immigrant populations. A study from Ireland found that non-native residents were less likely to attend a general practitioner than Irish-born individuals [37]. Similarly, studies from Norway and Spain reported significantly lower consultation rates among older immigrants compared with native populations [38,39].

Lower consultation frequencies among immigrants may reflect better overall health and therefore a reduced need for medical attention. However, this explanation was not supported by the administrative health data used in our study, and adjustment for health factors resulted in only minimal changes in the estimates. Furthermore, a Danish study found that immigrants from the Middle East, Northern Africa and Eastern Europe self-reported poorer health than natives [30].

The lower consultation frequency observed for some immigrant groups, as well as the high odds of non-utilisation across most immigrant categories, may be influenced by cultural differences in help-seeking behaviour, differing perceptions of which symptoms require medical attention, limited integration into the full scope of Danish primary care or variations in health literacy [40,41].

In contrast to our findings, a Norwegian study found that older adult immigrants from high-income countries used primary care services less frequently than native Norwegians [42], whereas we observed higher total consultation frequencies among immigrants from high-income countries compared with native Danes. A study based on data from 11 European countries, including Denmark, reported that immigrants aged ≥50 years had a significantly higher number of general practice visits than the native populations. Adjustment for health and socioeconomic factors reduced these differences but did not fully explain them [43]. These findings contradict our results for Eastern European immigrants, which showed significantly lower total consultation frequencies than natives. The discrepancy may be due to methodological differences, as the European study relied on survey data in which participants self-reported the number of general practice visits over a 12-month period. Part of the discrepancy may relate to the different age groups studied, as the European analysis included adults from 50 years upward, whereas our results apply specifically to immigrants aged 75 years and older. Additionally, although the European study used variables similar to those used in our study, it incorporated country-level variables based on the country of residence rather than the country of origin.

The reduced utilisation of Danish general practice services among older immigrant populations may reflect underlying inequities in access to health care. Older immigrants in Denmark may utilise various out-of-hours and acute health care services for non-urgent health issues, potentially due to limited understanding of the organisation of the Danish health care system and insufficient guidance on appropriate service utilisation.

Lastly, it is important to recognise the challenges associated with the 2018 amendment requiring patients with more than three years of residency in Denmark to pay a user fee for professional interpretation services in health care. A Danish qualitative study found a substantial decrease in the use of professional interpreters in general practice after the fee was introduced [44]. Instead, patients increasingly relied on family members for interpretation or attended consultations without an interpreter. This impaired mutual understanding between patients and GPs and resulted in suboptimal care for vulnerable patients. In addition, GPs faced ethical and legal challenges due to communication barriers [44]. Language barriers are among the known factors associated with poorer quality of and access to health care services [45]. The financial barrier may also disproportionately affect individuals with lower income, lower educational attainment and poorer health status, that is, groups who may have a greater need for professional interpretation in health care settings.

### Implications for further research and clinical practice

As the immigrant population continues to grow [15], health care planning and resource allocation may need to be reconsidered. Some immigrant groups may not utilise general practice sufficiently to benefit from available primary care services. In contrast, immigrants from Western countries demonstrated higher rates of general practice use compared with natives, suggesting increased demand from this group. Developing targeted educational initiatives about the structure and organisation of the Danish health care system may facilitate integration and help address underutilisation among certain groups, while simultaneously supporting the management of increased demand from Western-origin immigrants.

Language barriers and the role of professional interpretation in general practice warrant further investigation, particularly given that the existing user fee structure may contribute to inequity in access.

When patients forgo professional interpretation due to financial or other constraints, the quality of clinical communication may be compromised, raising the risk of misunderstandings and diagnostic errors. GPs should also be encouraged to develop greater awareness of the distinct vulnerabilities that some older immigrants face, with the aim of delivering more equitable care and reducing the impact of social barriers on health-seeking behaviour.

Further studies are needed to examine whether reduced general practice utilisation among older immigrants is associated with poorer morbidity and/or mortality outcomes, and to evaluate whether increased consultation frequency could yield clinical benefit. Finally, a deeper understanding of the patterns and determinants of immigrants’ use of out-of-hours health care services remains an important area for further investigation.

## Supplementary Material

Supplemental Material

## Data Availability

All data are available from Statistics Denmark and The Danish Health Data Authority upon application.
