# Supplementary material for: Older immigrants’ use of general practice in Denmark a register-based cohort study
Source: Scand J Prim Health Care. 2026 May 11;44(1):2666625. doi: 10.1080/02813432.2026.2666625 (PMC13162557; doi:10.1080/02813432.2026.2666625)
Supplement: Supplemental Material [file IPRI_A_2666625_SM8176.docx]

**Supplementary materials**

| **Supplementary Material 1 (30)** | |
| --- | --- |
| **Geographic region** | **Countries included** |
| Danish background | Denmark |
| Other Western background | Andorra, Australia, Austria, Belgium, Bulgaria, Canada, Croatia, Cyprus, Czech Republic, Estonia, Finland, France, Germany, Greece, Hungary, Iceland, Ireland, Italy, Latvia, Liechtenstein, Lithuania, Luxembourg, Malta, Monaco, the Netherlands, New Zealand, Norway, Poland, Portugal, Romania, San Marino, Slovakia, Slovenia, Spain, Sweden, Switzerland, United States of America, Vatican City, and United Kingdom |
| Eastern European background | Albania, Armenia, Azerbaijan, Bosnia and Herzegovina, Georgia, Belarus, Kazakhstan, Kosovo, Macedonia, Moldova, Montenegro, Russia, Serbia, Ukraine, former Soviet Union, and former Yugoslavia |
| Middle Eastern or Northern African background | Algeria, Bahrain, Egypt, United Arab Emirates, Iraq, Iran, Israel, Jordan, Kuwait, Lebanon, Libya, Morocco, Oman, Palestine, Qatar, Saudi-Arabia, Syria, Tunisia, Türkiye, and Yemen |
| Sub-Saharan African background | Angola, Benin, Botswana, Burkina Faso, Burundi, Cameroon, Cape Verde, the Central African republic, Congo, the Comoros, Djibouti, Côte d'Ivoire, Eritrea, Eswatini, Ethiopia, Equatorial Guinea, Gabon, Gambia, Ghana, Guinea, Guinea-Bissau, Kenya, Lesotho, Liberia, Madagascar, Malawi, Mali, Mauritania, Mauritius, Mozambique, Namibia, Niger, Nigeria, Rwanda, São Tomé and Príncipe, Senegal, the Seychelles, Sierra Leone, Somalia, Sudan, South Africa, South Sudan, Tanzania, Tchad, Togo, Uganda, Zambia, and Zimbabwe |
| Central or Southern American background | Antigua and Barbuda, Argentina, Bahamas, Barbados, Belize, Brazil, Bolivia, Chile, Colombia, Costa Rica, Cuba, Dominica, the Dominican Republic, Ecuador, El Salvador, Grenada, Guatemala, Guyana, Haiti, Honduras, Jamaica, Mexico, Nicaragua, Panama, Paraguay, Peru, Saint Kitts and Nevis, Saint Vincent and the Grenadines, Suriname, Trinidad and Tobago, Uruguay, and Venezuela |
| Asian or Oceanian background | Afghanistan, Bangladesh, Bhutan, Brunei, Cambodia, China, Cook Islands, Fiji, India, Indonesia, Japan, Kyrgyzstan, Kiribati, Laos, Malaysia, Maldives, Mongolia, Myanmar, Nauru, Nepal, North Korea, Pakistan, Palau, Papua New Guinea, Philippines, Solomon Islands, Samoa, Singapore, Sri Lanka, South Korea, Tajikistan, Taiwan, Thailand, Tibet, Timor-Leste, Tonga, Turkmenistan, Tuvalu, Uzbekistan, Vietnam, and Vanuatu |

| **Supplementary Material 2** | | |
| --- | --- | --- |
| **Former country** | **Successor states** | **Country used for data representation** |
| Soviet Union | Armenia, Azerbaijan, Belarus, Estonia, Georgia, Kazakhstan, Kyrgyzstan, Latvia, Lithuania, Moldova, Russia, Tajikistan, Turkmenistan, Ukraine, Uzbekistan | Russia |
| Yugoslavia | Bosnia and Herzegovina, Croatia, Macedonia, Montenegro, Serbia, Slovenia | Serbia |
| Czechoslovakia | Czech Republic, Slovakia | Czech Republic |

| **Supplementary Material 3. Definition of variables** | | |
| --- | --- | --- |
| **Variable** | **Categories** | **Groups** |
| (I) Individual health factors: |  |  |
| Age | 5-year intervals from 75-100+ | 6 |
| Sex | Dichotomised | 2 |
| Multimorbidity | Divided into no multimorbidity (score ≤0), lowest, middle, and highest tertile. Prescriptions and diagnoses were included from respectively 6 months and 5 years prior to index date. | 4 |
| Polypharmacy | Calculated as redeeming more than 5 different prescription drugs (on fourth level of ATC code) during the last 120 days and categorised into four groups 0, 1-4, 5-9, and 10+ unique prescription drugs. Excluding antibiotics (J01 & P01BA01). | 4 |
| Level of home health care services | Divided into five categories: None, lowest, middle, and highest tertile, and nursing home resident. The tertiles was based on allocated time per week by the municipality in minutes during the year prior to the index date. | 5 |
| (II) Individual geographic factors: |  |  |
| Rural or urban district | Dichotomised into urban and rural. Rural defined as less than 200 inhabitants in a community and more than 200 meters between houses. | 2 |
| Travel distance by road from home address to the general practice | Categorised based on the remuneration structure for home visits (<4km, 4-20km, and ≥21km) | 3 |
| Travel distance by road to appointed emergency department | <10km, 10-25km, and ≥25km | 3 |
| (III) Individual socioeconomic factors: |  |  |
| Cohabitation | Divided into single or cohabitating at 31st of December the year prior to the index date. | 2 |
| Household income | Divided by number of cohabitants in the household and split into quintiles for the study population and used as ordinal categorical values for the year prior to the index date. | 5 |
| Household wealth | Divided by number of cohabitants in the household and split into quintiles for the study population and used as ordinal categorical values for the year prior to the index date. | 5 |

| **Supplementary Material 4** | | | | |
| --- | --- | --- | --- | --- |
| **Consultation type (service number Danish name)** | **Face-to-face** | **Phone** | **Email** | **Home visit** |
| Consultation (0101 Konsultation) | X |  |  |  |
| Annual chronic care consultation (0120 Årsstatus/årskontrol) | X |  |  |  |
| Consultation by telephone (0201 Telefonkonsultation) |  | X |  |  |
| Planned consultation by telephone (0204 Aftalt telefonkonsultation) |  | X |  |  |
| Social medicine telephone consultation (3201 Socialmedicinsk telefonkonsultation) |  | X |  |  |
| Assessment of patients at nursing home  (0124 Lægefaglig vurdering af patienter på kommunal akutfunktion/akutteam) |  |  |  | X |
| Consultation by email (0105 E-konsultation) |  |  | X |  |
| Consultation with municipality services by email (0127 E-konsultation med kommunen) |  |  | X |  |
| Consultation by video (0125 Videokonsultation) | X |  |  |  |
| Video conference (0126 Videokonference) | X |  |  |  |
| Talk therapy (6101 Samtaleterapi) | X |  |  |  |
| Home-vist (04*1 Sygebesøg *dækker alle sygebesøg uanset 3. tal i koden) |  |  |  | X |
| Preventive home-visit (0121 Opsøgende hjemmebesøg) |  |  |  | X |
| **§2 services** | **Face-to-face** | **Phone** | **Email** | **Home visit** |
| Home-visit after hospital admission, Capital region (4250 Opfølgende hjemmebesøg) |  |  |  | X |
| Annual dementia consultation, Capital region (4282 Kontrol demens (årlig)) | X |  |  |  |
| Communication with municipality acute functions, Capital region (4469 Kommunikation med kommunal akutfunktion) |  |  |  | X |
| Home-visit after hospital admission, Region of Southern Denmark (4176 Opfølgende hjemmebesøg) |  |  |  | X |
| Consultation after hospital admission in the clinic, Region of Southern Denmark (4676 Opfølgning efter indlæggelse) | X |  |  |  |
| Prevention of acute hospitalisation or non-beneficial rehospitalisation, Region of Southern Denmark (4442-4448 Forebyggelse af akutte indlæggelser eller uhensigtsmæssigt genindlæggelser) |  |  |  | X |
| Crisis counseling, Region of Southern Denmark (4609 Krisesamtale) | X |  |  |  |
| Course of talk therapy, Region of Southern Denmark (4610 Samtaleterapiforløb) | X |  |  |  |
| Home-visit after hospital admission, Central region (4213 Opfølgende hjemmebesøg) |  |  |  | X |
| Home-visit for terminal patient, Central region (4801 Terminalydelsen) |  |  |  | X |
| Assessment of patients by acute team, Central region (4449 Akutfunktion) |  |  |  | X |
| **Temporary COVID-19 services** | **Face-to-face** | **Phone** | **Email** | **Home visit** |
| Consultation by video (4436 Videokonsultation) | X |  |  |  |
| Laboratory test results by telephone if indicated (4483 Telefonisk kontakt til patienter om prøvesvar, såfremt det er fagligt indiceret) |  | X |  |  |
| Negative laboratory test results by email if indicated (4484 Svarafgivelse på email til patienter med negativt prøvesvar, hvor det er fagligt indiceret) |  |  | X |  |
| Renumeration code for service 0101 or 0120 provided by telephone according to national agreement (1002 Corona-registreringsydelse til ydelser som er aftalt kan udføres telefonisk) |  | X |  |  |
| Renumeration code for service 0120 or 6101 provided by video according to national agreement (1003 Corona-registreringsydelse til ydelser som er aftalt kan udføres på video) | X |  |  |  |

| **Supplementary Material 5. Consultation type distribution and odds of non-utilisation in general practice across categories stratified by characteristics of country of origin^1^ among citizens aged ≥75 years in Denmark in 2021** | | | | | | | | | | | |
| --- | --- | --- | --- | --- | --- | --- | --- | --- | --- | --- | --- |
|  | **Most prevalent country** | **Total** | | **Face-to-face** | | **Telephone** | | **Email** | | **Home visit** | |
| **Characteristics of country of origin** |  | IRR* | 95% CI | IRR* | 95% CI | IRR* | 95% CI | IRR* | 95% CI | IRR* | 95% CI |
| Danish, High, >10%, <200 | Denmark | Reference | | Reference | | Reference | | Reference | | Reference | |
| Other Western, High, 6-10%, <200 | Poland | 1.02 | (1.01;1.03) | 1.02 | (1.00;1.04) | 1.07 | (1.05;1.09) | 0.97 | (0.94;1.00) | 1.02 | (0.96;1.07) |
| Other Western, High, >10%, <200 | Norway | 1.07 | (1.06;1.08) | 1.01 | (1.00;1.02) | 1.07 | (1.05;1.08) | 1.09 | (1.07;1.11) | 0.98 | (0.94;1.02) |
| Other Western, High, >10%, ≥200 | Germany | 1.04 | (1.04;1.05) | 1.01 | (0.99;1.02) | 1.03 | (1.02;1.04) | 1.10 | (1.08;1.12) | 1.09 | (1.05;1.13) |
| Eastern European, Middle, 6-10%, <200 | Bosnia and Herzegovina | 0.82 | (0.71;0.75) | 0.87 | (0.85;0.90) | 0.90 | (0.87;0.93) | 0.98 | (0.93;1.02) | 0.82 | (0.74;0.90) |
| Middle Eastern / Northern African, Middle, <6%, <200 | Türkiye | 0.84 | (0.83;0.85) | 0.90 | (0.88;0.93) | 0.93 | (0.91;0.95) | 0.89 | (0.86;0.93) | 0.87 | (0.81;0.94) |
| Middle Eastern / Northern African, Middle, 6-10%, <200 | Iran | 1.01 | (0.99;1.04) | 1.06 | (1.02;1.10) | 1.08 | (1.04;1.13) | 0.82 | (0.77;0.88) | 1.01 | (0.89;1.15) |
| Asian / Oceanian, Middle, <6%, ≥200 | Pakistan | 0.83 | (0.86;0.89) | 0.91 | (0.88;0.94) | 0.94 | (0.92;0.97) | 0.84 | (0.80;0.88) | 0.92 | (0.84;1.00) |
|  | | | | | | | | | | | |
| **Characteristics of country of origin** |  | OR * | 95% CI | OR * | 95% CI | OR* | 95% CI | OR* | 95% CI | OR* | 95% CI |
| Danish, High, >10%, <200 | Denmark | Reference | | Reference | | Reference | | Reference | | Reference | |
| Other Western, High, 6-10%, <200 | Poland | 1.06 | (0.95;1.18) | 1.08 | (0.99;1.18) | 1.03 | (0.94;1.13) | 0.99 | (0.92;1.08) | 1.04 | (0.91;1.19 |
| Other Western, High, >10%, <200 | Norway | 0.99 | (0.91;1.08) | 0.99 | (0.92;1.06) | 0.93 | (0.87;1.00) | 0.85 | (0.80;0.90) | 0.88 | (0.79;0.97 |
| Other Western, High, >10%, ≥200 | Germany | 1.09 | (1.01;1.17) | 1.04 | (0.98;1.11) | 1.07 | (1.01;1.14) | 0.95 | (0.90;1.01) | 0.98 | (0.89;1.08) |
| Eastern European, Middle, 6-10%, <200 | Bosnia and Herzegovina | 1.46 | (1.27;1.67) | 1.52 | (1.36;1.71) | 1.44 | (1.27;1.62) | 1.75 | (1.53;1.99) | 1.17 | (0.95;1.45) |
| Middle Eastern / Northern African, Middle, <6%, <200 | Türkiye | 1.49 | (1.34;1.66) | 1.59 | (1.46;1.74) | 1.11 | (1.01;1.23) | 1.65 | (1.49;1.83) | 1.36 | (1.15;1.60) |
| Middle Eastern / Northern African, Middle, 6-10%, <200 | Iran | 1.31 | (1.06;1.60) | 1.19 | (1.01;1.42) | 1.07 | (0.88;1.29) | 1.17 | (0.97;1.40) | 1.05 | (0.78;1.42) |
| Asian / Oceanian, Middle, <6%, ≥200 | Pakistan | 1.95 | (1.74;2.18) | 1.96 | (1.77;2.17) | 1.43 | (1.28;1.59) | 1.74 | (1.54;1.95) | 1.24 | (1.02;1.50) |
| *^1^Characteristics of country of origin refer to country‑level attributes of each individual’s country of birth. These include geographical region, national income level, national health expenditure, and population density, which together define the categories presented in the table.*  *Order of characteristics: geographical region, income level, health expenditure, population density.*  *Low-income countries are not represented in the table, as the number of immigrants from these countries was insufficient to meet the inclusion criterion of ≥500 immigrants per category. IRR = Incidence rate ratio; OR = Odds ratio; CI = Confidence interval.  *Adjusted for age, sex, multimorbidity, polypharmacy, level of home health care services, rural or urban district, travel distance from home address to general practice, travel distance to appointed emergency department, cohabitation, household income, and household wealth.* | | | | | | | | | | | |

| **Supplementary Material 6.** **Hierarchical sensitivity analysis of total consultations in general practice across categories stratified by characteristics of country of origin^1^ among citizens aged ≥75 years in Denmark in 2021** | | | | | | | | | |
| --- | --- | --- | --- | --- | --- | --- | --- | --- | --- |
|  | **Most prevalent country** | **Crude** | | **Adjusted for health factors*** | | **Adjusted for health and geographic factors**** | | **Adjusted for health, geographic, and socioeconomic factors***** | |
| **Characteristics of country of origin** |  | IRR | 95% CI | IRR | 95% CI | IRR | 95% CI | IRR | 95% CI |
| Danish, High, >10%, <200 | Denmark | Reference | | Reference | | Reference | | Reference | |
| Other Western, High, 6-10%, <200 | Poland | 1.00 | (0.99;1.02) | 1.02 | (1.01;1.04) | 1.02 | (1.02;1.03) | 1.02 | (1.01;1.03) |
| Other Western, High, >10%, <200 | Norway | 1.07 | (1.06;1.07) | 1.08 | (1.07;1.09) | 1.07 | (1.07;1.08) | 1.07 | (1.06;1.08) |
| Other Western, High, >10%, ≥200 | Germany | 1.02 | (1.02;1.03) | 1.05 | (1.04;1.05) | 1.05 | (1.04;1.05) | 1.04 | (1.04;1.05) |
| Eastern European, Middle, 6-10%, <200 | Bosnia and Herzegovina | 0.82 | (0.80;0.83) | 0.81 | (0.80;0.83) | 0.81 | (0.79;0.82) | 0.82 | (0.80;0.83) |
| Middle Eastern / Northern African, Middle, <6%, <200 | Türkiye | 0.87 | (0.85;0.88) | 0.84 | (0.82;0.85) | 0.83 | (0.82;0.84) | 0.84 | (0.83;0.85) |
| Middle Eastern / Northern African, Middle, 6-10%, <200 | Iran | 1.01 | (0.98;1.03) | 1.01 | (0.99;1.04) | 1.00 | (0.98;1.03) | 1.01 | (0.99;1.04) |
| Asian / Oceanian, Middle, <6%, ≥200 | Pakistan | 0.84 | (0.83;0.85) | 0.83 | (0.82;0.84) | 0.82 | (0.81;0.84) | 0.83 | (0.82;0.85) |
|  | | | | | | | | | |
| **Characteristics of country of origin** |  | OR | 95% CI | OR | 95% CI | OR | 95% CI | OR | 95% CI |
| Danish, High, >10%, <200 | Denmark | Reference | | Reference | | Reference | | Reference | |
| Other Western, High, 6-10%, <200 | Poland | 1.08 | (0.97;1.20) | 1.06 | (0.96;1.18) | 1.10 | (0.98;1.22) | 1.05 | (0.95;1.18) |
| Other Western, High, >10%, <200 | Norway | 0.95 | (0.88;1.04) | 0.96 | (0.88;1.04) | 0.97 | (0.89;1.05) | 0.99 | (0.91;1.08) |
| Other Western, High, >10%, ≥200 | Germany | 1.12 | (1.05;1.20) | 1.10 | (1.02;1.18) | 1.10 | (1.02;1.18) | 1.09 | (1.01;1.17) |
| Eastern European, Middle, 6-10%, <200 | Bosnia and Herzegovina | 1.66 | (1.45;1.89) | 1.59 | (1.39;1.82) | 1.67 | (1.46;1.91) | 1.46 | (1.27;1.67) |
| Middle Eastern / Northern African, Middle, <6%, <200 | Türkiye | 1.77 | (1.60;1.96) | 1.61 | (1.45;1.78) | 1.70 | (1.53;1.88) | 1.49 | (1.34;1.66) |
| Middle Eastern / Northern African, Middle, 6-10%, <200 | Iran | 1.61 | (1.32;1.96) | 1.40 | (1.14;1.71) | 1.48 | (1.20;1.81) | 1.31 | (1.06;1.60) |
| Asian / Oceanian, Middle, <6%, ≥200 | Pakistan | 2.15 | (1.93;2.40) | 2.02 | (1.81;2.27) | 2.14 | (1.91;2.40) | 1.95 | (1.74;2.18) |
| *^1^Characteristics of country of origin refer to country‑level attributes of each individual’s country of birth. These include geographical region, national income level, national health expenditure, and population density, which together define the categories presented in the table.*  *Order of characteristics: geographical region, income level, health expenditure, population density.*  *Low-income countries are not represented in the table, as the number of immigrants from these countries was insufficient to meet the inclusion criterion of ≥500 immigrants per category.  IRR = Incidence rate ratio; OR = Odds ratio; CI = Confidence interval*  **Health factors = Age, sex, multimorbidity, polypharmacy, level of home health care services*  ***Geographic factors = Rural or urban district, travel distance from home address to general practice, travel distance to appointed emergency department*  ****Socioeconomic factors = Cohabitation, household income, household wealth* | | | | | | | | | |

| **Supplementary Material 7. Distribution of consultation types, measured as mean and median, across categories stratified by characteristics of country of origin^1^ among citizens aged ≥75 years in Denmark in 2021** | | | | | | | | | | |
| --- | --- | --- | --- | --- | --- | --- | --- | --- | --- | --- |
|  | **Total** | | **Face-to-face** | | **Telephone** | | **Email** | | **Home visit** | |
| **Characteristics of country of origin** | Mean | Median | Mean | Median | Mean | Median | Mean | Median | Mean | Median |
| Danish, High, >10%, <200 | 10.06 | 7  (2-14) | 4.18 | 3  (0-6) | 3.25 | 2  (0-4) | 2 | 0  (0-2) | 0.54 | 0  (0-0) |
| Other Western, High, 6-10%, <200 | 9.84 | 7  (2-14) | 3.94 | 2  (0-6) | 3.41 | 2  (0-5) | 2 | 0  (0-2 | 0.52 | 0  (0-0) |
| Other Western, High, >10%, <200 | 10.76 | 8  (2-15) | 4.14 | 3  (0-6) | 3.54 | 2  (0-5) | 3 | 0  (0-3) | 0.57 | 0  (0-0) |
| Other Western, High, >10%, ≥200 | 10.19 | 7  (2-15) | 4.12 | 3  (0-6) | 3.24 | 2  (2-4) | 2 | 0  (0-2) | 0.56 | 0  (0-0) |
| Eastern European, Middle, 6-10%, <200 | 7.42 | 4  (1-11) | 2.97 | 1  (0-5) | 2.74 | 1  (0-4) | 1 | 0  (0-0) | 0.37 | 0  (0-0) |
| Middle Eastern / Northern African, Middle, <6%, <200 | 7.88 | 5  (1-12) | 3.05 | 1  (0-5) | 3.14 | 2  (0-4) | 1 | 0  (0-1) | 0.38 | 0  (0-0) |
| Middle Eastern / Northern African, Middle, 6-10%, <200 | 9.27 | 6  (1-14) | 3.90 | 2  (0-6) | 3.59 | 2  (0-5) | 1 | 0  (0-1) | 0.43 | 0  (0-0) |
| Asian / Oceanian, Middle, <6%, ≥200 | 7.20 | 4  (0-11) | 2.78 | 1  (0-4) | 2.84 | 1  (0-4) | 1 | 0  (0-0) | 0.37 | 0  (0-0) |
| *^1^Characteristics of country of origin refer to country‑level attributes of each individual’s country of birth. These include geographical region, national income level, national health expenditure, and population density, which together define the categories presented in the table.*  *Order of characteristics: geographical region, income level, health expenditure, population density.*  *Low-income countries are not represented in the table, as the number of immigrants from these countries was insufficient to meet the inclusion criterion of ≥500 immigrants per category.*  *Parentheses indicate the 25th–75th percentile (p25–p75).* | | | | | | | | | | |
